# A Novel Method for Detecting Intersectional DIF: Multilevel Random Item Effects Model with Regularized Gaussian Variational Estimation

**DOI:** 10.1017/psy.2025.10046

**Published:** 2025-09-15

**Authors:** He Ren, Weicong Lyu, Chun Wang, Gongjun Xu

**Affiliations:** 1 College of Education, University of Washington, Seattle, WA, USA; 2 Faculty of Education, University of Macau, Macau, China; 3 Department of Statistics, University of Michigan, Ann Arbor, MI, USA

**Keywords:** differential item functioning, intersectional DIF, regularization, variational estimation

## Abstract

Differential item functioning (DIF) screening has long been suggested to ensure assessment fairness. Traditional DIF methods typically focus on the main effects of demographic variables on item parameters, overlooking the interactions among multiple identities. Drawing on the intersectionality framework, we define intersectional DIF as deviations in item parameters that arise from the interactions among demographic variables beyond their main effects and propose a novel item response theory (IRT) approach for detecting intersectional DIF. Under our framework, fixed effects are used to account for traditional DIF, while random item effects are introduced to capture intersectional DIF. We further introduce the concept of intersectional impact, which refers to interaction effects on group-level mean ability. Depending on which item parameters are affected and whether intersectional impact is considered, we propose four models, which aim to detect intersectional uniform DIF (UDIF), intersectional UDIF with intersectional impact, intersectional non-uniform DIF (NUDIF), and intersectional NUDIF with intersectional impact, respectively. For efficient model estimation, a regularized Gaussian variational expectation-maximization algorithm is developed. Simulation studies demonstrate that our methods can effectively detect intersectional UDIF, although their detection of intersectional NUDIF is more limited.

## 1 Introduction

The heavy reliance on assessments in critical social decision-making, such as college admission, personnel selection and placement, and resource allocation, highlights the need for a thorough evaluation of assessment fairness, particularly in light of ongoing concerns about equity. For decades, assessment fairness has been a central focus in psychometrics. American Educational Research Association et al. (2014) further emphasizes the importance of ensuring assessment fairness throughout the test development process, including the standard practice of screening for differential item functioning (DIF).

DIF refers to the phenomenon in which people from different subgroups, usually defined by demographic variables, such as gender, race, or ethnicity, differ in the probability of correctly answering an item after controlling for their ability. Although DIF does not necessarily indicate measurement bias, DIF detection is a critical first step for further investigation. Two types of DIF are often discussed in the literature: uniform DIF (UDIF) and non-uniform DIF (NUDIF). Specifically, UDIF assumes a consistent difference in item responses between groups across ability levels, whereas NUDIF allows this difference to vary across ability levels. Various DIF detection methods have been developed, including Lord’s chi-square test, logistic regression, and regularized DIF (Lord, 1980; Swaminathan & Rogers, 1990; Tutz & Schauberger, 2015; Wang et al., 2023). While these methods differ in many ways, they typically treat DIF as the main effect of each demographic variable.

Recently, two criticisms have emerged concerning the quantitative methodologies used in inequality studies, including those employed in DIF analysis. First, existing methods often overlook intersectionality. In reality, people’s multiple identities do not function in isolation but are interlinked to collectively shape the privilege and discrimination. Intersectionality, a theoretical framework rooted in feminist scholarship, highlights this complexity and is increasingly used in fields, such as health, psychology, and education studies (Cole, 2009; Núñez, 2014). In the context of DIF, this framework gives rise to the concept of intersectional DIF, which refers to the DIF that results from the interaction effect of multiple demographic variables. Unlike traditional DIF, which only considers the main effect of demographic variables separately, intersectional DIF captures the potential bias that arises at the intersection of multiple identities. For example, individuals belonging to multiple marginalized groups may experience DIF effects that are not simply the sum of the effect of each grouping variable, but amplified or diminished due to their intersecting social positions. Empirical results from a recent intersectional DIF study suggest that traditional DIF methods that ignore intersectionality may lead to substantial bias (Albano et al., 2024). Second, existing studies often require the specification of a reference group. DIF is usually detected by comparing each focal group to the reference group, while comparisons among focal groups themselves are rarely made. Although mathematically any group can be designated as the reference with no difference, the routine choice of the privileged group may unintentionally reinforce the notion that privileged groups represent the norm, positioning all other groups as deviations (Johfre & Freese, 2021).

In response to these concerns, recent studies have begun to address intersectional DIF (Albano et al., 2024; Belzak, 2023; Muthén & Asparouhov, 2018; Russell & Kaplan, 2021; Russell et al., 2021, 2022). These methods typically model intersectional DIF by either incorporating both demographic variables and their interactions (i.e., product terms) into the measurement model, or by defining a single synthetic categorical variable that encodes all combinations of demographic characteristics. The synthetic group method is mathematically equivalent to modeling all-way interactions. However, both methods treat intersectional DIF as fixed effects, which limits scalability. As more demographic variables are included, the number of intersectional groups and corresponding parameters increases geometrically, while the sample size per group decreases. This leads to challenges for model estimation. For example, the combination of gender (e.g., male, female, and non-binary) and race (e.g., White, Black, or African American, American Indian or Alaska Native, Asian, and Native Hawaiian or Other Pacific Islander) results in 15 intersectional groups, and this number expands rapidly as additional variables are considered.

In contrast to traditional fixed-effect methods for DIF detection, intersectionality can be modeled as random effects within the multilevel modeling framework. This approach is inspired by multilevel analysis of individual heterogeneity and discriminatory accuracy (MAIHDA), an emerging quantitative approach developed in health inequality. MAIHDA treats individuals (level 1) as nested within intersectional strata (level 2), where each stratum represents a unique combination of social identities, that is, a specific level of the synthetic intersectional group variable. MAIHDA models incorporate the main effect for each demographic variable and a stratum-level random effect. Rather than modeling all-way interaction terms explicitly through fixed effects, the random effect captures the total between-strata variance that is not explained by the additive main effects. Compared to traditional fixed-effect methods, this multilevel framework promotes model parsimony and scalability in the presence of many demographic variables and enables the decomposition of covariate effects into additive and interactive components (Evans et al., 2024; Evans et al., 2018; Merlo, 2018). It is worth noting that, in MAIHDA, the same demographic variables that characterize individuals at level 1 also define the level 2 strata. While this seems to introduce collinearity, there is a conceptual distinction. As clarified in the MAIHDA literature, unlike in conventional multilevel models where demographic variables are treated as individual-level covariates, these variables are conceptualized as properties of the strata at level 2. This framing is fundamental to the MAIHDA framework and is discussed in detail by Evans et al. (2024).

Similar to the MAIHDA framework, we propose applying random effects to item parameters for detecting intersectional DIF. In this approach, the main effects of demographic variables on item parameters are explicitly modeled to account for traditional DIF, while random effects are introduced to capture additional variations across intersectional groups without requiring the explicit specification of interaction terms. A random item effect with nonzero variance, after controlling for main effects, is interpreted as evidence of intersectional DIF. Using random effects, the proposed model inherits the advantages of MAIHDA, including interpretability, scalability, and parsimony.

Although this multilevel approach is new to DIF detection, it builds on the well-known random item effect framework in psychometrics. Specifically, random-item item response theory (IRT) models allow item parameters to vary across groups following specific distributions. These models have been applied in various measurement invariance contexts, such as longitudinal designs with randomly drawn item samples, international large-scale assessments, and automatic item generation (AIG) or item cloning (De Boeck, 2008; Jong et al., 2007; Lathrop & Cheng, 2017; Muthén & Asparouhov, 2018; Rijmen & Jeon, 2013). However, existing random item effect models cannot be used directly for intersectional DIF detection. First, most existing models define groups using a single demographic variable (e.g., country) and do not involve the decomposition of main and interaction effects. When extended to intersectional groups formed by multiple identities, using only random effects confounds interactions with main effects. In other words, without explicitly modeling main effects, the random effect cannot be directly interpreted as intersectional DIF (Jong et al., 2007; Muthén & Asparouhov, 2018; Rijmen & Jeon, 2013). Second, existing models typically assume random effects on all items, requiring post hoc tests to identify DIF items. Third, model estimation is computationally intensive. Although Rijmen & Jeon (2013) employ variational inference to reduce computational efforts, their algorithm still lacks closed-form solutions and remains computationally demanding.

Our proposed methods address these limitations through three innovations. First, as mentioned above, the proposed models incorporate both main effects of demographic variables and random effects, enabling separation between traditional and intersectional DIF. Second, we impose a log penalty on the random item effects, effectively shrinking the variance to zero for items free from intersectional DIF. Notably, due to this regularization, our methods do not require anchors for random effects. However, anchor items are still needed for main effects, as we assume that both traditional and intersectional DIF can appear on the same item. Since the primary focus of this study is on intersectional DIF, the anchor requirement applies only to main effects and thus plays a limited role. Third, for efficient model estimation, we develop a Gaussian variational expectation–maximization (GVEM) algorithm. Originally introduced to psychometrics for multidimensional IRT (MIRT) estimation, GVEM circumvents the high-dimensional integral in model estimation, achieves a closed-form solution within the EM algorithm, and significantly reduces computational complexity (Cho et al., 2021).

Our methods accommodate both intersectional UDIF and NUDIF detection by applying a unified modeling strategy to different item parameters (i.e., difficulty and discrimination). In addition, we extend the model to capture intersectional impact. Impact refers to differences in the group-level mean abilities. Traditionally, impact is limited to the main effects of demographic variables on group-level ability means. However, as emphasized by intersectionality, interactions among multiple identities could also influence group-level abilities. We define intersectional impact as different group-level mean abilities arising from these interactions. Similar to intersectional DIF, we use random effects on group-level mean abilities (i.e., multilevel latent trait) to capture intersectional impact. Beyond studying intersectionality, our proposed approach is also well suited for nested structures, such as students within different countries, especially in large-scale assessments (Pastor, 2003; Sulis & Toland, 2017).

In summary, the major contributions of this article are fourfold: (1) quantifying the intersectional DIF as random item effects, (2) introducing the concept of intersectional impact, (3) applying a log penalty to detect nonzero item-level variation reflective of intersectional DIF, and (4) applying efficient variational methods for model estimation. The rest of the article is organized as follows. We first introduce the four random item IRT models proposed in this study, followed by the regularized GVEM algorithm. Then, we present four simulation studies and an empirical study to evaluate the performance of the proposed intersectional DIF detection methods. Finally, we conclude with a discussion of limitations and future directions.

## 2 Methods

This study aims to detect intersectional DIF, defined as interactions among demographic variables on item parameters. We also consider scenarios both with and without intersectional impact, defined as interactions among demographic variables that affect group-level mean abilities. The two-parameter logistic (2PL) IRT model is used as the foundational model, upon which four extended models are developed. These models incorporate random item intercepts for intersectional UDIF, random item slopes for intersectional NUDIF, and a multilevel ability structure for intersectional impact. Specifically, the four proposed models are 2PL with random item intercept (2PL-Ri), 2PL with random item intercept and with multilevel latent trait (2PL-RiM), 2PL with random item intercept and slope (2PL-Ris), and 2PL with random item intercept and slope and with multilevel latent trait (2PL-RisM). The structure and applicability of these models are summarized in Table 1.

**Table 1:** Proposed IRT models in this study

	2PL-Ri	2PL-RiM	2PL-Ris	2PL-RisM
Random intercept	Yes	Yes	Yes	Yes
Random slope	No	No	Yes	Yes
Multilevel latent trait	No	Yes	No	Yes
DIF scenario	UDIF	UDIF with impact	UDIF and NUDIF	UDIF and NUDIF with impact

*Note*: DIF and impact refer to intersectional DIF and intersectional impact, respectively.

Let 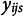 denote the binary response of person *i* ( 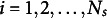 ) in group *s* ( 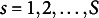 ) on item *j* ( 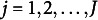 ). For 2PL-RisM, the most flexible model in this study, the item response function of 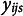 is (1)

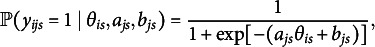

where the random effects are (2)

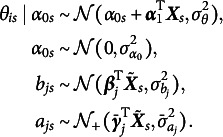

Before interpreting the model, it is necessary to clarify several notations. Let *D* denote the number of demographic variables of interest. An intersectional group is defined as a unique combination of levels across these *D* variables. Let *S* be the total number of such intersectional groups, equal to the product of the number of levels across all variables. That is, 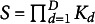 , where 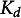 is the number of levels for the *d*-th variable. For each group 
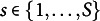
 , let 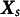 be a *P*-dimensional dummy coded vector. The total number of dummy variables is 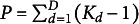 . For example, if there are 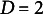 variables, race and gender, where race has 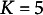 categories and gender has 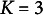 categories, then 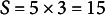 , and 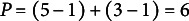 . To accommodate the intercept, let 
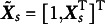
 be a 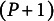 -dimensional vector. Correspondingly, 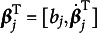 , where 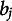 denotes the intercept parameter for item *j* in the reference group, and 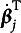 is the vector of coefficients representing the main effects of demographic variables on the intercept. Note that in the current model, one intersectional group serves as the reference group because demographic variables are dummy coded to capture main effects. However, by using effect coding instead, no single intersectional group is treated as the reference; rather, effects are interpreted relative to the overall mean across all groups. Similarly, 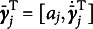 , where 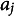 denotes the slope parameter for item *j* in the reference group, and 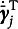 represents the main effects on the slope. Finally, 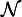 and 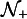 denote the normal distribution and the truncated normal distribution (left-truncated at zero), respectively. We place bars over parameters associated with 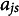 to indicate that 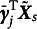 and 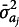 represent the mean and variance of the untruncated latent variable underlying 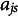 , rather than those of 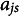 itself.

In Equation (1), 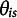 is the ability of person *i* in subgroup *s*, where abilities within each subgroup follow a normal distribution with mean 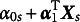 . Recall that 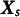 is the dummy coding vector that corresponds to group *s*. 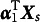 represents the main effect of demographics on the group-level mean ability, that is, the traditional impact in DIF literature. In addition, we introduce the random intercept 
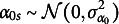
 . As in the MAIHDA literature, we do not explicitly model any high-order interactions among the demographic variables. Instead, the random effect 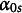 is used to capture these additional deviations, that is, the intersectional impact. Moreover, 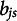 represents the group-specific intercept parameter for item *j* in subgroup *s*, following a normal distribution with mean 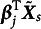 and variance 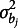 . The term 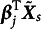 represents the group-specific intercept due to the main effects of demographic variables, corresponding to the traditional UDIF. The variance of the random intercept 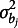 captures deviations from the main effect across intersectional groups and is intended to reflect intersectional DIF on the intercept. Similarly, the group-specific slope parameter 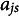 follows a truncated normal distribution. Its pre-truncation mean, 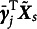 , captures the main effects of demographics on the slope (i.e., traditional NUDIF), while the variance 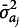 is specifically introduced to capture intersectional DIF on the slope. Accordingly, the model is parameterized so that item *j* is free of intersectional NUDIF when 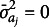 , and further free of intersectional UDIF when 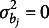 as well.

Please note that the 2PL-RisM model shown above combines features of the random item effect model and multilevel IRT model. It treats item parameters similarly to a linear logistic test model with error (LLTM with error), but instead of using a property matrix to explain the difficulty (De Boeck, 2008; Kim & Wilson, 2020), the mean of each item’s difficulties is determined by the main effects of demographics to capture traditional DIF, while the variance accounts for additional variations (i.e., intersectional DIF). In addition, the other three models in Table 1 are simplified version of the 2PL-RisM model: the 2PL-Ris model sets 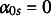 , the 2PL-RiM model sets 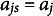 , and the 2PL-Ri model sets both 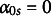 and 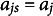 .

### 2.1 Model estimation

In this section, we introduce a novel algorithm for model estimation based on variational inference. The key idea of variational approximation is to approximate the intractable marginal likelihood with a computationally feasible lower bound. The lower bound derived in this article follows the local variational methods by Bishop (2006) and Cho et al. (2021). Compared to the variational methods by Rijmen & Jeon (2013), the GVEM method in this article results in closed-form solutions for most parameters. To ensure clarity, we begin with the simplest model in this study, 2PL-Ri. The item response function of 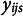 can still be written as in Equation (1), with random effects (3)

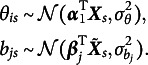



Let 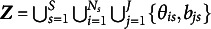 be the set of all latent variables, including both latent traits and random item effects, in the 2PL-Ri model. The joint likelihood of responses 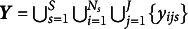 and latent variables 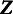 is (4)

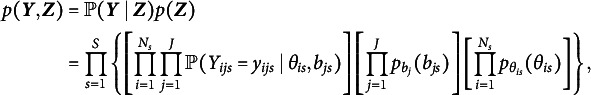

where 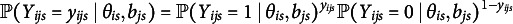 . With any probability density function 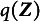 for 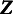 , the log marginal likelihood of 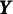 can be written as (5)

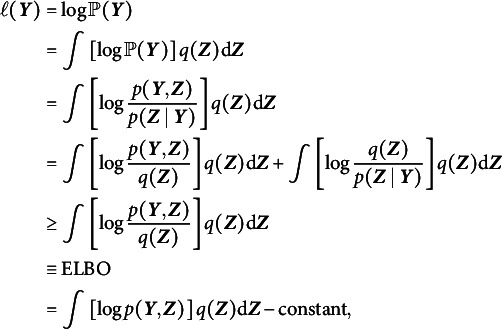

where ELBO refers to the evidence lower bound, and the difference 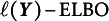 corresponds to the Kullback–Leibler (KL) divergence (Kullback & Leibler, 1951) from 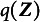 to 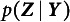 , given by 

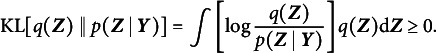

Note that the constant in Equation (5), 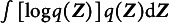 , depends only on *q* and can therefore be omitted from the optimization. Optimizing 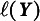 is thus reduced to maximizing 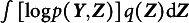 . The EM algorithm achieves this by setting 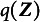 such that 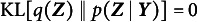 . In the E-step, it computes the expectation of the log-likelihood (i.e., 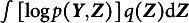 ). In the M-step, this expectation is maximized with respect to model parameters. However, the regular EM algorithm requires that the expectation is computationally feasible, which hardly holds in the random item effect models. In the 2PL-Ri model, for example, the expectation in Equation (5) involves a high-dimensional integral with respect to 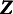 , a latent variable of dimension 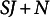 , where 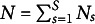 is the total sample size. We address this challenge by applying variational inference for estimation.

In the context of 2PL-Ri, given Equations (1) and (3)–(4), we have (6)

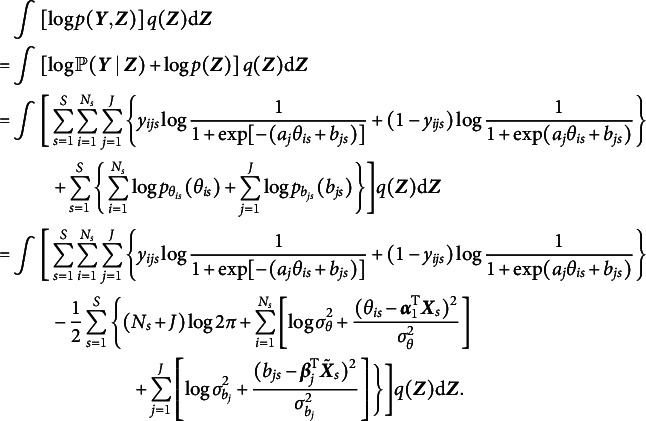

The difficulty in computing the marginal log-likelihood in Equation (6) primarily arises from the sigmoid function, which prevents closed-form integration. We adopted a local variational method (Bishop, 2006) to approximate the sigmoid function with a computationally feasible lower bound. As demonstrated in Cho et al. (2021), a sigmoid function can be expressed as (7)

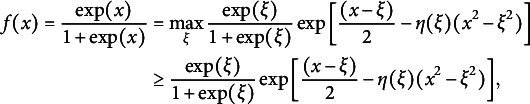

where 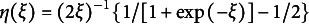 , and 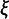 is the variational parameter used to approximate the sigmoid function, which is updated iteratively in the EM algorithm. Applying Equation (7) to Equation (6), we obtain (8)

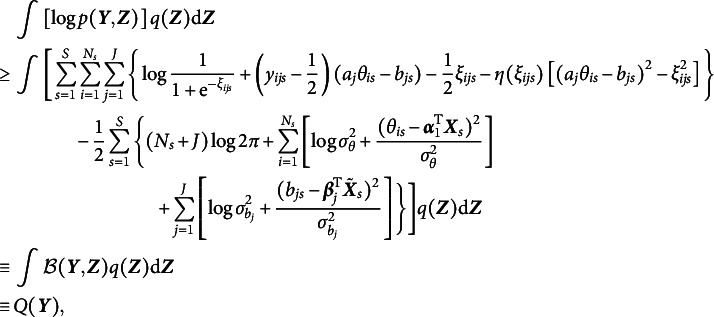

where 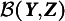 denotes a lower bound of 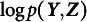 under the local variational approximation.

Next, we need to determine a variational density 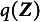 that closely approximates the true posterior 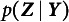 , such that 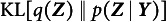 is minimized. This ensures that the ELBO provides a tight approximation to the marginal log-likelihood 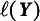 , as shown in Equation (5). Under the mean-field variational assumption (Bishop, 2006), we approximate the posterior distribution of the latent variables using a product of independent factors, each corresponding to a separate latent variable, i.e., 

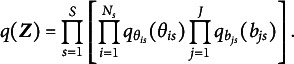

Note that the latent variables in 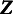 need not be truly independent, as the goal is to approximate its true posterior distribution while simplifying the computation. Then, for any latent variable 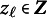 , its optimal variational distribution 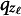 takes the form 

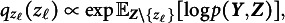

where 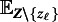 refers to the expectation over all latent variables in 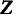 other than 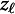 (Bishop, 2006; Blei et al., 2017). With the lower bound 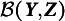 , we update the variational distribution as 

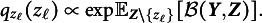

Thus, the optimal 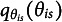 and 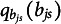 that maximize the ELBO (i.e., minimize the KL divergence) are given by (9)

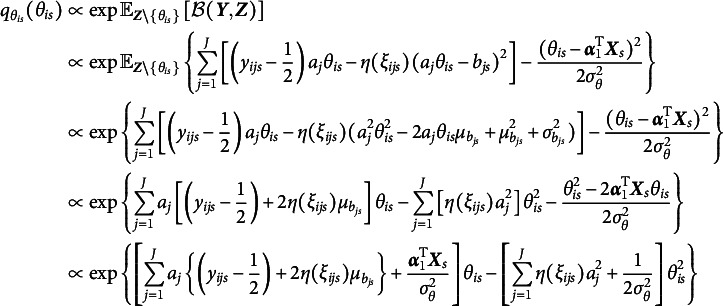

and (10)

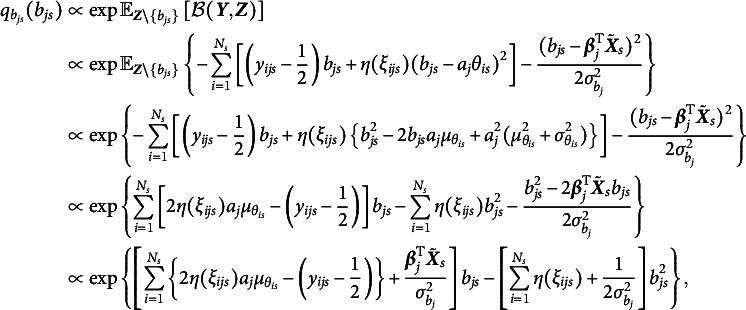

respectively. As shown in Equation (9), the variational density of 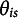 is an exponential family with sufficient statistics 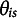 and 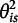 , and thus 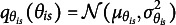 , where (11)

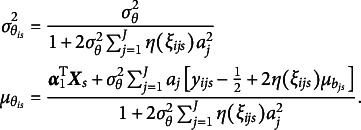

Similarly, the variational density of 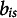 shown in Equation (10) also follows a normal distribution, that is, 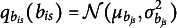 , where (12)

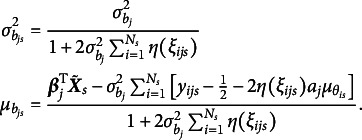

Given the optimal variational distributions derived above and the mean-field variational assumption, we compute the expectation over all latent variables with respect to the variational distribution 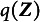 in Equation (8), yielding (13)

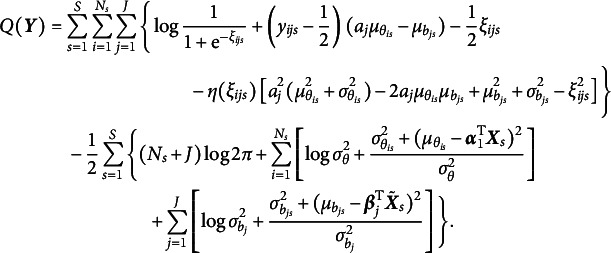



In addition, a log penalty is imposed on 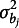 in Equation (13) to encourage sparsity in item random effects for intersectional DIF detection. The log penalty has been employed for identifying permissible attribute patterns in cognitive diagnostic models (Gu & Xu, 2019; Ma et al., 2023; Wang, 2024). Note that 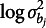 is already included in 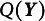 . On the one hand, incorporating the log penalty preserves closed-form solutions in the M-step, thereby ensuring computational efficiency. On the other hand, as shown by Ma et al. (2023), the log penalty has a Bayesian interpretation: it corresponds to placing a Dirichlet prior with parameter 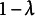 on the variances. When 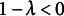 , the prior becomes an improper Dirichlet distribution, which promotes the selection of significant variances more aggressively than traditional proper Dirichlet priors. Overall, a regularized GVEM algorithm is proposed, where the objective function to be maximized is given by 

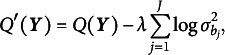

where 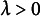 is a tuning parameter and larger values of 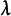 result in greater sparsity in 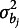 .

In each EM iteration, variational densities in Equations (11) and (12) are updated in the E-step. In the M-step, 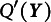 is maximized to update all model parameters. This is achieved by setting the derivative of the objective function with respect to each model parameter to be zero. We will show that all parameters of the 2PL-Ri model can be updated with closed-form solutions, leading to a computationally efficient algorithm. We fix 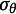 to 1 for model identification, and the update rules for all other model parameters are presented below: (14)

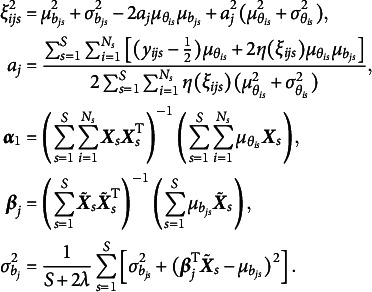

Following the derivation shown above, similar variational lower bounds can be derived for the other three proposed models. The detailed derivation for the most complex model in this study, 2PL-RisM, is provided in the Appendix.

Lastly, we employ the generalized information criterion (GIC) to select an appropriate value of 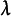 , as it has been shown to have desirable theoretical properties (Cho et al., 2024; Fan & Tang, 2013). Specifically, GIC takes the form of (15)

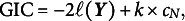

where *k* is the number of DIF parameters and 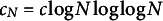 , with *c* being a constant that controls the degree of model sparsity. When 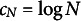 , GIC reduces to the Bayesian information criterion (BIC). Since 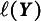 is computationally intractable due to high-dimensional integration, we instead use 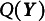 as a surrogate in Equation (15) to compute the GIC.

The regularized GVEM algorithm for DIF detection in the 2PL-Ri model is summarized in Algorithm 1. Two remarks are worth noting.

Remark 1. The log penalty term 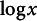 might lead to numerical instability when *x* approaches zero. Although 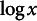 does not appear in the iterations of the GVEM algorithm as shown in Equations (11), (12), and (14), it is required for computing the GIC. Therefore, we replace 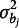 with 
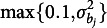
 in the GIC calculation whenever 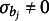 .

Remark 2. We do not penalize main DIF effects (i.e., 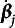 and/or 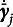 ) because the primary goal of this study is to detect intersectional DIF, which is defined through nonzero variance terms. As a result, anchor items must be prespecified, in contrast to approaches in the literature that penalize main effects directly (see, e.g., Wang et al., 2023). If an additional penalty term, such as the lasso, were imposed on these main effects, anchor items would no longer be required. In this study, we use four anchor items, corresponding to 20% of the total test length.

**Figure:**
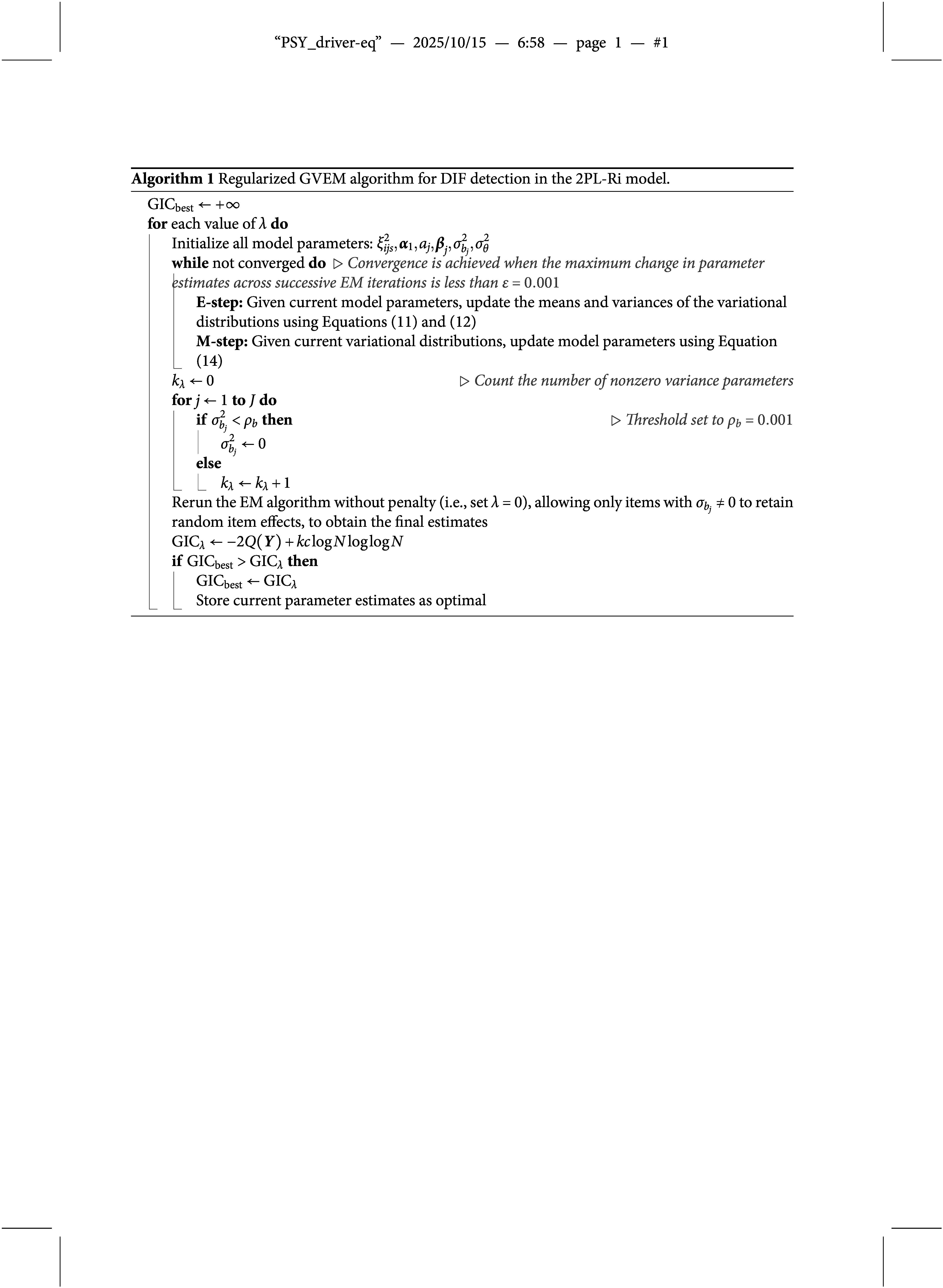

## 3 Simulation studies

Four simulation studies are conducted to evaluate the performance of the proposed regularized GVEM algorithm in detecting intersectional DIF. Studies I–IV corresponded to the four models, 2PL-Ri, 2PL-RiM, 2PL-Ris, and 2PL-RisM, respectively, each targeting at a different DIF scenario, as detailed in the Methods section. In all studies, the number of items is fixed to 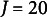 . Following Huang et al. (2024), the slope parameters 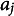 ( 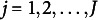 ) are drawn from 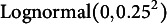 , and intercept 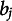 ( 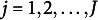 ) are drawn from 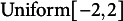 . The true item parameters are given in Table 2.

**Table 2:** True fixed item parameters for the simulation studies

Item	1	2	3	4	5	6	7	8	9	10
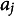	0.691	1.483	0.787	0.795	0.607	0.934	0.924	0.855	0.974	1.113
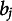	0.354	0.122	1.911	−1.209	1.377	−1.620	−0.475	−1.816	−1.390	1.099
Item	11	12	13	14	15	16	17	18	19	20
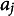	0.823	0.724	0.823	1.003	0.963	0.839	1.346	1.089	1.135	0.929
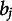	−0.422	−0.554	−0.316	−0.712	0.209	1.885	0.232	0.297	0.565	1.296

Each simulation study systematically manipulates four common factors. First, the number of intersectional groups is set to either 10 or 40. For the 10-group conditions (i.e., 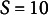 ), groups are defined by two demographic variables, one binary (e.g., sex) and one five-category variable (e.g., occupational status), resulting in 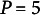 for the dummy-coded variables. For the 40-group conditions, groups are defined by four demographic variables, three binary (e.g., sex, immigrant background, and dichotomous education level) and one five-category variable (e.g., occupational status), resulting in 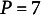 for the dummy-coded variables. This setup aligns with an empirical study on intersectionality (Keller et al., 2023). Second, the sample size per group is set to either 50 or 100. Third, the proportion of items with intersectional DIF is set at 20% (Items 1–4) or 60% (Items 1–12). Intersectional DIF is introduced by nonzero random item effects. For items with intersectional UDIF, half are assigned 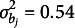 and the other half 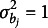 . For items with intersectional NUDIF, half are assigned 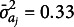 and the other half 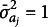 . These magnitudes are derived from a pilot study using PISA data, where the variances for intersectional DIF ranged from 0.31 to 0.98 for intercepts (centered at 0.54) and from 0.22 to 0.46 for slopes (centered at 0.33). All items with intersectional NUDIF include both random intercept and random slope, reflecting real-world scenarios where NUDIF often coexists with UDIF (Wang et al., 2023). Fourth, traditional impact, defined as mean ability differences due to the main effects by demographic variables, is either absent or present. When present, the traditional impact is set at 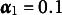 , yielding ability mean differences ranging from 0.1 to 0.2 for the 10-group conditions and up to 0.4 for the 40-group conditions.

Beyond the four common factors, Studies II and IV also consider intersectional impact. This is introduced by the variance of the group-level random intercept on ability ( 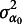 ), set to either 0 (absence) or 0.5 (presence). These values correspond to intra-class correlation (ICC) values of approximately 0 and 0.1, aligning with the ICC in empirical intersectional educational assessment literature (Keller et al., 2023). Overall, Studies I and III included 16 experimental conditions each, while Studies II and IV included 32 conditions each. A summary of all manipulated factors is provided in Table 3.

**Table 3:** Illustration of simulation designs

	Simulation I	Simulation II	Simulation III	Simulation IV
	(2PL-Ri)	(2PL-RiM)	(2PL-Ris)	(2PL-RisM)
Number of groups (*S*)	10, 40	10, 40	10, 40	10, 40
Sample size per group ( 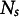 )	50, 100	50, 100	50, 100	50, 100
Proportion of DIF items	20%, 60%	20%, 60%	20%, 60%	20%, 60%
Traditional impact ( 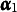 )	0.1, 0	0.1, 0	0.1, 0	0.1, 0
Intersectional impact ( 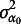 )	—	0.5, 0	—	0.5, 0

We note again that this study focuses on detecting intersectional DIF, rather than traditional DIF. To avoid confounding due to traditional DIF and to demonstrate the models’ ability to disentangle traditional and intersectional DIF, all items are designed to exhibit traditional DIF, which is introduced through fixed main effects of demographic variables. More specifically, we set 
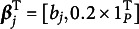
 and 
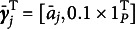
 in Equation (2). Under the 10-group conditions, this setup results in traditional DIF magnitudes ranging from 0.2 to 0.4 for intercepts and 0.1 to 0.2 for slopes across groups. For the 40-group conditions, these ranges increase to 0.2 to 0.8 for intercepts and 0.1 to 0.4 for slopes, following the design by Belzak & Bauer (2020). To ensure model identification in the presence of traditional DIF across all items, 20% of items (Items 17–20) are designated as anchors with main effects fixed at zero (i.e., 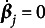 and 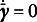 ). However, their random effects are still freely estimated, meaning that they are not anchored with respect to intersectional DIF.

The flagging procedure for intersectional DIF has been shown in Algorithm 1. Each condition is replicated 50 times, and the performance is measured by false positive (FP) and true positive (TP) rates. Specifically, the FP rate refers to the proportion of items free from intersectional DIF mistakenly flagged as DIF items, while the TP rate refers to the proportion of items with intersectional DIF that are correctly detected. We consider 50 replications sufficient since the TP and FP rates are averaged across all the DIF-free and DIF-related item parameters, rather than being evaluated for a single parameter in each replication.

### 3.1 Simulation I: UDIF detection

**Figure 1:**
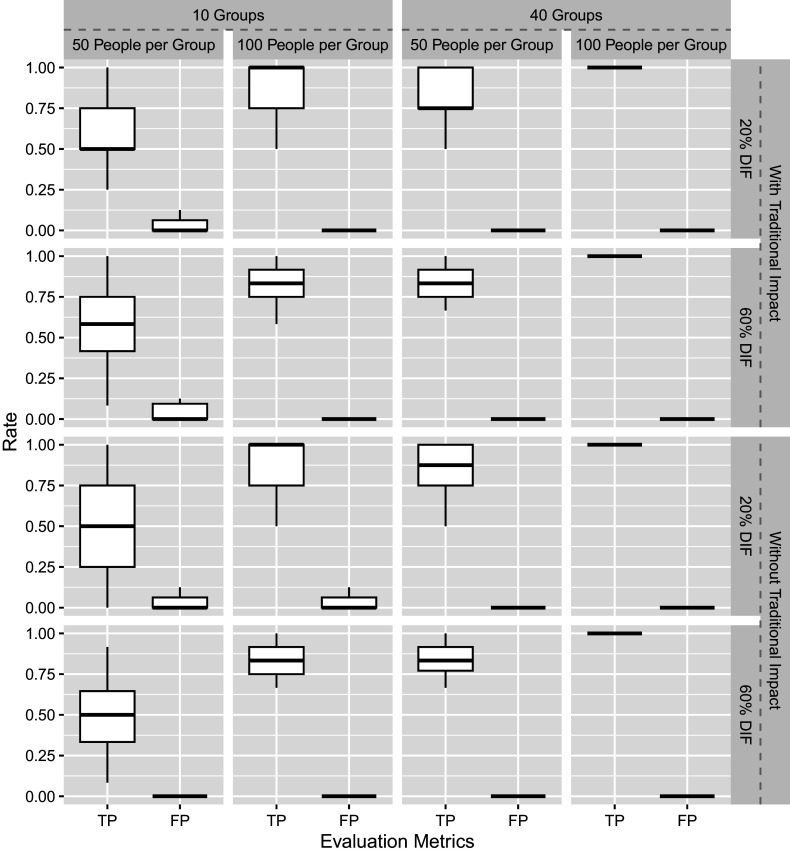
Simulation I results.

We evaluate 2PL-Ri in this simulation, where slope parameters are fixed across groups. Figure 1 shows the TP and FP rates of Simulation I across 50 replications. Under most conditions, except when 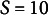 and 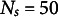 , the new method performs well. Overall, the 2PL-Ri model performs better with more groups and larger sample sizes per group. As intersectional DIF is modeled by random effects, such results are not surprising but consistent with the findings from the multilevel modeling literature (Adam et al., 2021; Maas & Hox, 2005; Moineddin et al., 2007). In addition, the proportion of DIF items and the presence or absence of intersectional impact have minimal influence on the results.

### 3.2 Simulation II: UDIF detection with intersectional impact

**Figure 2:**
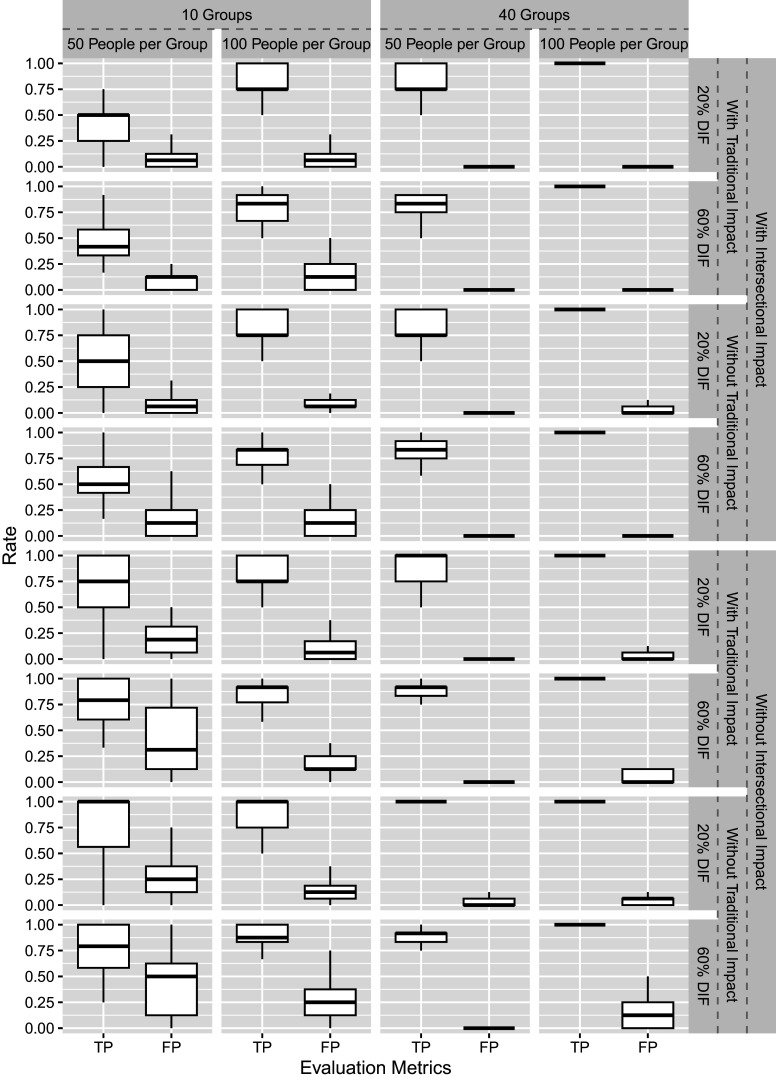
Simulation II results.

We study 2PL-RiM in Simulation II, where intersectional impact is considered. As shown in Figure 2, the new method follows a pattern similar to Simulation I. That is, the proposed method performs well under most conditions except when 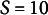 and 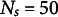 , and unsurprisingly, it performs better with more groups and larger sample sizes per group. In addition, the proportion of DIF items has a small effect on performance, with a lower proportion yielding slightly better results. Similarly, the presence or absence of traditional and intersectional impact has minimal influence on the results.

### 3.3 Simulation III: UDIF and NUDIF detection

**Figure 3:**
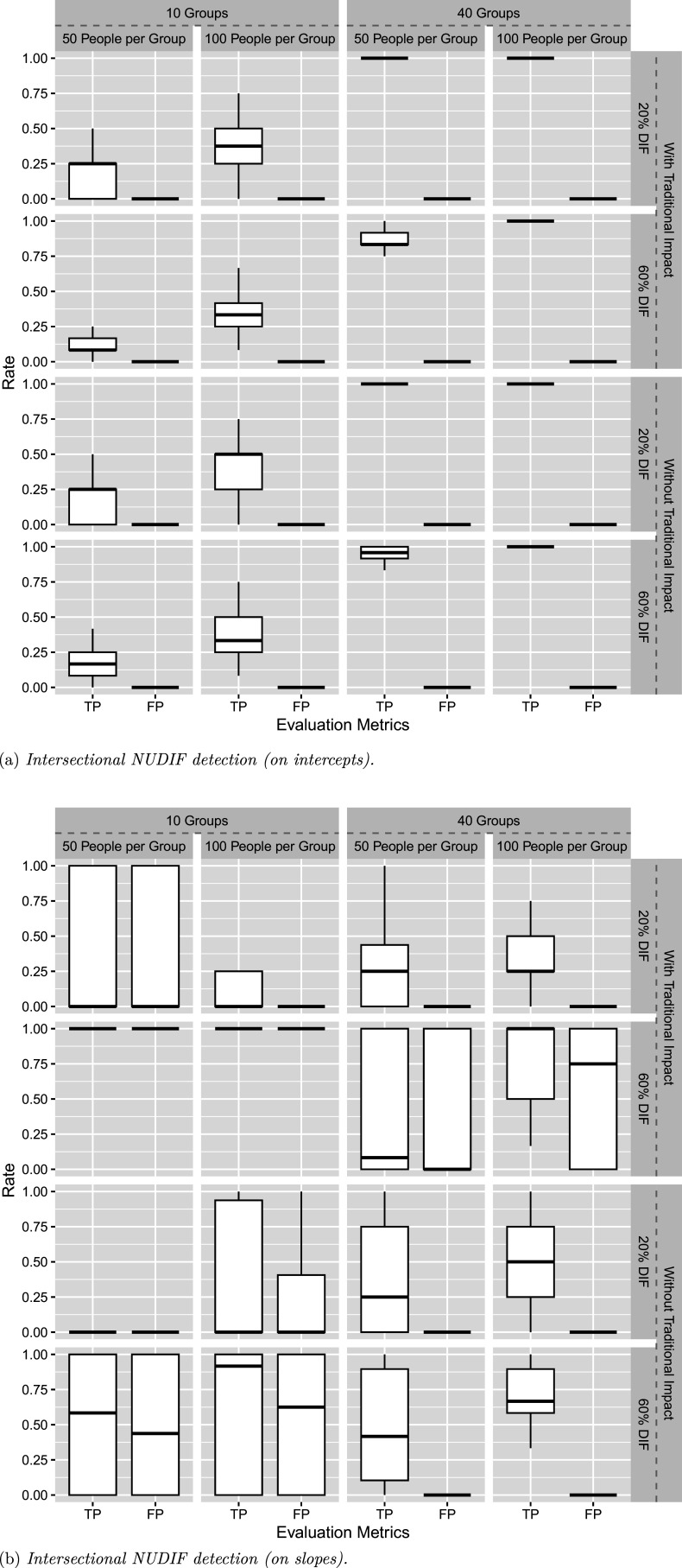
Simulation III results.

2PL-Ris is evaluated with both UDIF and NUDIF incorporated. Figure 3a and 3b summarizes the DIF detection results on intercept and slope parameters, respectively. With intersectional NUDIF, the proposed method maintains desirable performance on intercepts with 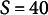 while resulting in worse performance with the smaller group number 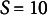 . The DIF detection results for slope parameters are generally unsatisfactory. Relatively better performance was observed under conditions involving a large number of groups and either the absence of traditional impact or the combination of the presence of traditional impact and a low proportion of DIF items. In general, this is consistent with prior studies, which found that it is more challenging to identify DIF effects on slope parameters than on intercepts (Bauer et al., 2020; Wang et al., 2023). Regarding the manipulated factors, while the number of groups and the sample size per group have consistent effects across Simulations I–III, the influence of DIF proportion and traditional impact becomes more pronounced in this study. Lastly, compared to Simulation I, DIF detection results for the intercept exhibit lower TP rates when 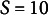 . Additional guidance on the use of this method is provided in the Discussion section.

### 3.4 Simulation IV: UDIF and NUDIF detection with intersectional impact

**Figure 4:**
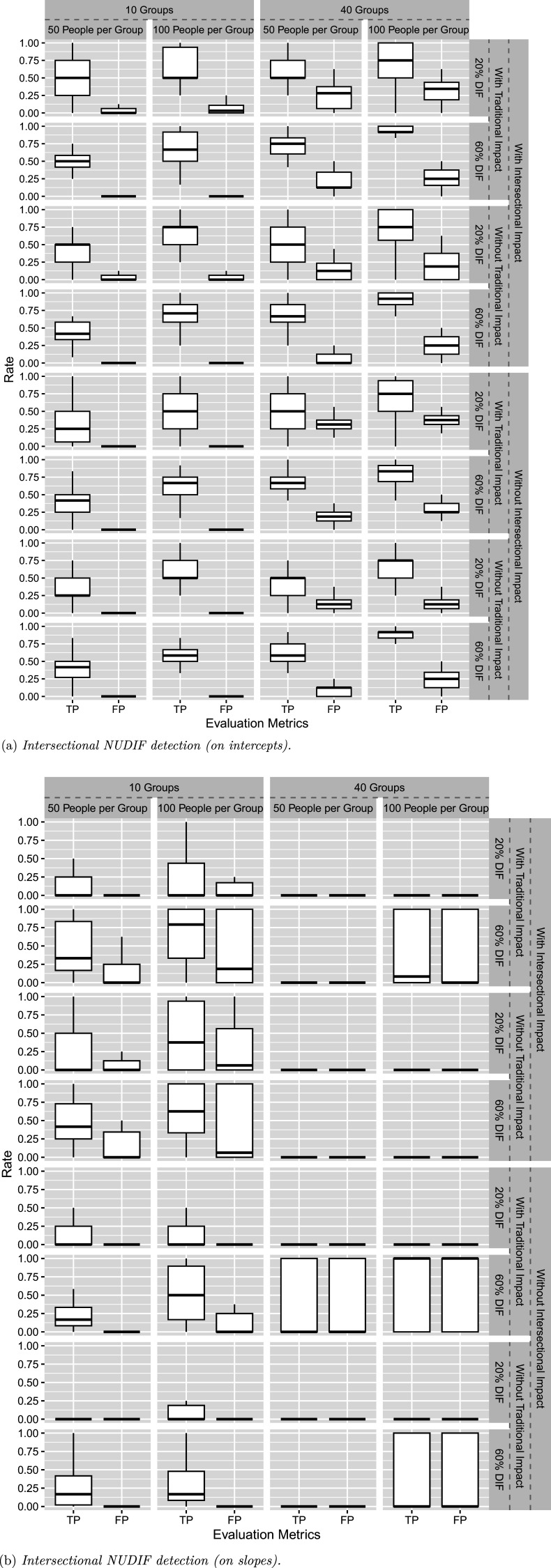
Simulation IV results.

The final simulation study evaluates the 2PL-RisM model, with the TP and FP rates summarized in Figure 4. In general, the method results in desirable TP and FP rates for detecting intersectional UDIF, but performs poorly in detecting intersectional NUDIF. In fact, the NUDIF detection results are generally unacceptable across nearly all conditions.

## 4 Empirical study

A real data set from the Programme for International Student Assessment (PISA) is used to demonstrate the performance of the four methods in this article. PISA is a well-known international large-scale assessment that tests the skills and knowledge of 15-year-old students in mathematics, reading, and science (OECD, 2019). In this study, we use a subset of the PISA 2018 science assessment, including dichotomous responses of 7,002 students on 19 items. Three demographic variables are considered: (1) country (eight countries in the subset), (2) sex (male or female), and (3) highest parental education (below or at least college level). These variables are chosen due to their frequent consideration in studies on educational equity. The full combination of these variables results in 32 intersectional groups, with their corresponding sample sizes summarized in Table 4.

**Table 4:** Sample size for each group in the empirical study

	Below college			At least college	
	Male	Female		Male	Female
ALB	175	120		93	94
ARE	300	233		951	927
AUS	258	282		536	522
AUT	110	133		184	189
BEL	115	98		255	293
BGR	71	62		100	111
BIH	68	68		53	86
BLR	38	35		205	237

**Figure 5:**
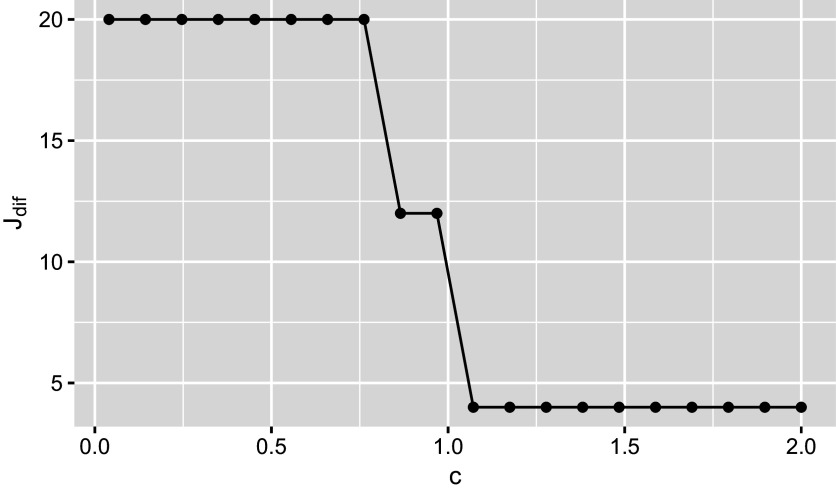
Relationship between the number of items exhibiting intersectional DIF and *c*.

Before discussing our empirical findings, we introduce a feasible way to tune the hyperparameter *c* in GIC (Lyu et al., 2025). Figure 5 illustrates the procedure, where *c* is plotted against 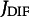 , the number of items exhibiting intersectional DIF. Similar to the scree plots in factor analysis, Figure 5 suggests that the models chosen by GIC with 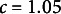 , which corresponds to the “elbow” of the plot. In practice, the choice of *c* can also depend on research goals. In certain high-stakes testing contexts, a higher FP rate may be acceptable in order to achieve a high TP rate, as undetected DIF can lead to serious fairness concerns.

The empirical data set is analyzed using each of the four models, and the results are summarized in Table 5, where *a* and *b* refer to (intersectional) NUDIF and UDIF, respectively. Items 7, 17, 18, and 19 are flagged as UDIF items by most models, and no item is flagged as NUDIF.

**Table 5:** DIF detection results of the empirical study

	1	2	3	4	5	6	7	8	9	10	11	12	13	14	15	16	17	18	19
2PL-Ri							*b*										*b*	*b*	*b*
2PL-RiM					*b*		*b*										*b*	*b*	*b*
2PL-Ris		*b*					*b*										*b*	*b*	*b*
2PL-RisM		*b*	*b*	*b*															

*Note*: *b* indicates UDIF, and NUDIF is not detected.

Given the simulation results indicating unsatisfactory performance in detecting NUDIF, we focus our empirical analysis on the results from the 2PL-Ri and 2PL-RiM models. To validate the empirical results, we estimate the RiM and Ri model using Markov chain Monte Carlo (MCMC) via the rstan package (Stan Development Team, 2024), allowing random item effects only for the consistently flagged items (i.e., items 7, 17, 18, and 19). For comparison, we also fit a constrained version of the model without random item effects. Model comparison based on the leave-one-out information criterion (LOOIC) reveals that the model with random item effects provides a significantly better fit, with a LOOIC difference of 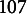 and a standard error of 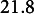 . Furthermore, for comparison, a total score-based method is also employed to examine intersectional DIF (Belzak, 2023). Specifically, Belzak’s method (2023) uses regularized logistic regression, with total score as the matching criterion and intersectionality modeled through interactions among demographic variables. This method is chosen due to its similarity to our proposed methods, as it accounts for both main and intersectional DIF effects with a primary focus on UDIF. However, this method has two limitations: (1) it does not automatically account for impact, since the total score is not directly regressed on demographic variables and (2) it may struggle with a large number of demographic variables, given that interactions are modeled using fixed effects. Despite these limitations, the method identifies UDIF in items 2, 5, 7, 14, 18, and 19, which largely aligns with the findings from our proposed methods.

## 5 Discussion

This study proposes a novel random effects IRT approach for detecting intersectional DIF and demonstrates the feasibility of applying a regularized GVEM algorithm in this context. By including both item-level and person-level random effects, the model accounts for intersectional DIF and impact effects arising from multiple demographic variables. Through the GVEM framework, all model parameters can be updated by closed-form solutions when detecting UDIF, resulting in a computationally efficient model estimation procedure. Simulation results show that the proposed methods can effectively detect UDIF. We have further extended the method to detect intersectional NUDIF, which is known to be more challenging. In this setting, all model parameters except the main and random effects on item discrimination (i.e., 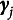 and 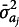 ) have closed-form solutions (see the Appendix for details). The simulation results reveal that the number of groups has the most substantial impact on performance, followed by the sample size per group, the proportion of DIF items, and the presence or absence of impact. In terms of computational efficiency, the method performs well on standard hardware. On a laptop with an Intel i7-12700H CPU, the runtimes for a typical setting (i.e., 20 items, 20% DIF items, 40 groups, and 100 people per group) with a single regularization parameter range from 7.23 to 12.41 seconds, depending on the model used. These results underscore the scalability of the proposed approach for large-scale assessments.

In this study, intersectional DIF is modeled using random effects, and variation in group sizes may influence the methods’ performance. Literature on multilevel modeling has shown that unequal cluster sizes can reduce both the power to detect true effects and the efficiency of estimating fixed and random components (Candel & Breukelen, 2009; Kush et al., 2022; Manatunga et al., 2001). Specifically, Candel & Breukelen (2009) found that the relative efficiency (RE) of the random intercept variance estimator can drop to between 84% and 95%, depending on the distribution and range of the cluster sizes. They also found that the loss in RE can be recovered by increasing the number of clusters, where the compensatory adjustment is given by 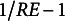 . For example, if the RE is 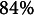 , then 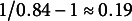 , suggesting that an increase of 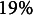 more clusters is needed to restore the original efficiency.

While we explore intersectional NUDIF detection alongside UDIF, the results for intersectional NUDIF detection are unsatisfactory, particularly when the model simultaneously accounts for intersectional impact. A supplementary simulation study demonstrates that even when response data are generated from the 2PL-Ris model, which includes intersectional NUDIF, the 2PL-Ri model still effectively identified items exhibiting intersectional UDIF. This suggests that, in practice, researchers should mainly rely on the detection results for intersectional UDIF when using the proposed methods. The results for intersectional NUDIF should be interpreted with caution and be used primarily in cases where intersectional impact is not included and when both the sample size and the number of groups are sufficiently large. Despite these challenges, our framework provides a foundation for future advances in intersectional NUDIF detection. In a pilot study where only random effects, but not main effects, were considered, the methods demonstrate better results for NUDIF detection, suggesting potential for improvement. Future studies could explore the incorporation of regularization in both random effects and main effects to overcome estimation challenges. In addition, modeling slope parameters with lognormal distributions instead of truncated normal distributions may offer further improvement.

Another limitation of the simulation studies is that how demographic variables affect the variance of ability is not considered. Specifically, while mean latent traits are allowed to vary across groups, the within-group variance is assumed to be constant. Although this assumption aligns with most DIF research, several educational studies, such as Baye & Monseur (2016) and Gray et al. (2019), have discovered differences in latent trait variances among demographic groups. Future research may explore how demographic variables influence the variance of latent traits.

This study employs a variational approach to approximate the log marginal likelihood. Although parameter estimation may be biased due to the use of a mean-field Gaussian distribution family to approximate the posterior distribution of the latent variables, the approximation becomes increasingly accurate with larger sample sizes. Nevertheless, the estimation of latent variables, including group-specific item parameters with random effects and person abilities, may not be sufficiently accurate. To address this issue, we propose applying the standard MCMC procedure for their estimation, as described in the empirical study. Alternatively, future research could consider using best linear unbiased prediction (BLUP) as a complementary or alternative approach to MCMC.

Lastly, it is important to recognize that DIF arises within a complex social context. Each individual carries a unique set of experiences that shape their learning and life trajectories. However, when patterns of advantage or disadvantage emerge at the group level, they serve as a reminder that systemic discrimination continues to persist. Thus, while detecting DIF is a crucial first step in examining issues of fairness, it must be followed by deeper investigations into the underlying causes of structural inequality.

## Data Availability

The code that supports the findings of this study will be available on the project webpage (https://sites.uw.edu/pmetrics/projects/) shortly as we are still working on creating user-friendly R package and Shiny App. The real data was downloaded directly from the PISA website.

## A Appendix

The derivation of the 2PL-RisM model estimation procedure is shown below. The model is presented in Equations (1) and (2). Applying the local variational method in Equation (7), we obtain

(A1)

Then, the corresponding optimal variational distributions for the latent variables are

where

where

where

where

Given the optimal variational distributions derived above and the mean-field variational assumption, the expectation (i.e., the integral) in Equation (A1) can be computed as

The objective function with log penalty is

By setting the derivative of the objective function with respect to each model parameter to be zero, we get the following parameters update rules:
